# The brain acylcarnitine profile depends on nutritional state

**DOI:** 10.1186/2193-1801-4-S1-P46

**Published:** 2015-06-12

**Authors:** Baiba Svalbe, Marina Makreck-Kuka, Eduards Sevostjanovs, Maija Dambrov, Edgars Liepinsh

**Affiliations:** Latvian Institute of Organic Synthesis, Latvia; Riga Stradins University, Latvia

**Keywords:** acylcarnitine, brain structures, nutritional state

In addition to glucose, also fatty acids are important as an energy source in the brain where about 20% of the energy is gained from mitochondrial oxidation of fatty acids. Fatty acids are transported into mitochondria in the form of acylcarnitine. It is known that acylcarnitine concentration in the heart and blood plasma varies depending on the nutritional state, fed or fasted. The aim of the present study was to compare the concentration of short-chain (C2-C5), medium-chain (C6-C12) and long-chain (C14-C18) acylcarnitines in fed and fasted states in rat brain structures: cerebellum, cortex and hypothalamus. The total concentration of acylcarnitines was not affected by nutrient state in none of brain structures studied, but we found that cortex contained less acylcarnitines than cerebellum and hypothalamus. The nutritional state did not affect the overall concentration of short-chain acylcarnitines in the brain structures. In contrary, the nutritional state significantly affected the concentration of total medium chain acylcarnitines in the cortex: 0.33 and 0.46nmol/g tissues in fed and fasted state, respectively. The highest concentration of medium-chain acylcarnitines was found in hypothalamus in fed and fasted state compared to cortex and cerebellum. The nutritional state significantly affected the concentration of total long-chain acylcarnitines in the cortex: 4.92 and 5.88nmol/g tissues in fed and fasted state, respectively. In fed state the highest concentrations of long-chain acylcarnitines was found in hypothalamus compared to cortex and cerebellum. The results demonstrate that the nutrition state affects brain acylcarnitine concentration in different brain structures, and cortex is the most affected brain structure. Further studies are needed to investigate the role of changes in acylcarnitine profile in the brain signalling pathways.

